# THE NEUTROPHIL-LYMPHOCYTE RATIO AND WHITE BLOOD CELL COUNT ASSOCIATE WITH MUSCLE ENERGETICS AND GAIT SPEED IN SOMMA

**DOI:** 10.1093/geroni/igae098.1721

**Published:** 2024-12-31

**Authors:** Philip Kramer, Peggy Cawthon, Li-Yung Lui, Theodore DeConne, Nancy Lane, Xiaoyan Leng, Anne Newman, Stephen Kritchevsky

**Affiliations:** Wake Forest University School of Medicine, Winston-Salem, North Carolina, United States; California Pacific Medical Center, San Francisco, California, United States; California Pacific Medical Center, San Francisco, California, United States; Wake Forest University School of Medicine, Winston-Salem, North Carolina, United States; University of California Davis School of Medicine, Davis, California, United States; Wake Forest University School of Medicine, Winston-Salem, North Carolina, United States; University of Pittsburgh, University of Pittsburgh, Pennsylvania, United States; Wake Forest University School of Medicine, Wake Forest University School of Medicine, North Carolina, United States

## Abstract

Systemic inflammation drives sarcopenia and cachexia, which impair the mobility and independence of older adults. We and others have demonstrated that muscle mitochondrial energetics are strongly associated with gait speed, leg power, and mobility limitations. However, the contribution of systemic inflammation to lower muscle mitochondrial energetics is not fully understood. In this analysis of the Study of Muscle, Mobility and Aging (SOMMA), two pro-inflammatory biomarkers, the neutrophil-lymphocyte ratio (NLR) and total WBC count (WBC), were measured in adults aged 30-94 (30-69, n=81; 70-79, n=864). We report associations with maximal muscle mitochondrial energetics (MaxOXPHOS) and 400m gait speed. Individuals in higher NLR tertiles (< 1.685 vs. ≥1.685 - < 2.430 vs. ≥2.430) had significantly lower MaxOXPHOS (63.9±22.5 vs. 61.7±19.9 vs. 59.0±16.8, p=0.019) and gait speed (1.08±0.19 vs. 1.08±0.18 vs. 1.03±0.16, p=0.0002), had a greater multimorbidity burden (p < 0.0001), and were older (p=0.008) compared to individuals in lower NLR tertiles. The associations of NLR and WBC with MaxOXPHOS (NLR, β=-1.38, p=0.05; WBC, β=-2.45, p=0.0003) were partly explained by age and BMI (NLR, ∆β=-37%, p=0.18; WBC, ∆β=-34%, p=0.01), and were further explained when accounting for all other covariates, including multimorbidity burden (NLR, ∆β=-28%, p=0.36; WBC, ∆β=-43%, p=0.15). However, the association of NLR and WBC with gait speed (NLR, β=-0.019, p=0.0009; WBC, β=-0.034, p< 0.0001) could not be fully explained by age, BMI or other covariates (NLR, ∆β=-10.5%, p=0.002; WBC, ∆β=-61%, p=0.019). In conclusion, systemic inflammation, especially as related to age, obesity, and disease, associates with lower muscle energetics and gait speed in adults.

